# Expanding the clinical spectrum of *COL1A1* mutations in different forms of glaucoma

**DOI:** 10.1186/s13023-016-0495-y

**Published:** 2016-08-02

**Authors:** Lucia Mauri, Steffen Uebe, Heinrich Sticht, Urs Vossmerbaeumer, Nicole Weisschuh, Emanuela Manfredini, Edoardo Maselli, Mariacristina Patrosso, Robert N. Weinreb, Silvana Penco, André Reis, Francesca Pasutto

**Affiliations:** 1 Medical Genetics, A.O. Niguarda Ca’Granda Hospital, Milan, Italy; 2 Institute of Human Genetics, Friedrich-Alexander-Universität Erlangen-Nürnberg (FAU), Erlangen, Germany; 3 Bioinformatics, Institute of Biochemistry, FAU Erlangen-Nürnberg, Erlangen, Germany; 4 Augenklinik und Poliklinik, Joahnnes Gutenberg-Universität Mainz, Mainz, Germany; 5 Molecular Genetics Laboratory, Institute for Ophthalmic Research, Centre for Ophthalmology, University of Tuebingen, Tuebingen, Germany; 6 Clinica Zucchi, Monza, Italy; 7 Shiley Eye Institute, UC San Diego, San Diego, CA USA

**Keywords:** *COL1A1*, Congenital glaucoma, Early onset glaucoma, Osteogenesis imperfecta, Whole exome sequencing

## Abstract

**Background:**

Primary congenital glaucoma (PCG) and early onset glaucomas are one of the major causes of children and young adult blindness worldwide. Both autosomal recessive and dominant inheritance have been described with involvement of several genes including *CYP1B1, FOXC1, PITX2, MYOC* and *PAX6*. However, mutations in these genes explain only a small fraction of cases suggesting the presence of further candidate genes.

**Methods:**

To elucidate further genetic causes of these conditions whole exome sequencing (WES) was performed in an Italian patient, diagnosed with PCG and retinal detachment, and his unaffected parents. Sanger sequencing of the complete coding region of *COL1A1* was performed in a total of 26 further patients diagnosed with PCG or early onset glaucoma. Exclusion of pathogenic variations in known glaucoma genes as *CYP1B1, MYOC, FOXC1, PITX2* and *PAX6* was additionally done per Sanger sequencing and Multiple Ligation-dependent Probe Amplification (MLPA) analysis.

**Results:**

In the patient diagnosed with PCG and retinal detachment, analysis of WES data identified compound heterozygous variants in *COL1A1* (p.Met264Leu; p.Ala1083Thr). Targeted *COL1A1* screening of 26 additional patients detected three further heterozygous variants (p.Arg253*, p.Gly767Ser and p.Gly154Val) in three distinct subjects: two of them diagnosed with early onset glaucoma and mild form of osteogenesis imperfecta (OI), one patient with a diagnosis of PCG at age 4 years. All five variants affected evolutionary, highly conserved amino acids indicating important functional restrictions. Molecular modeling predicted that the heterozygous variants are dominant in effect and affect protein stability and thus the amount of available protein, while the compound heterozygous variants act as recessive alleles and impair binding affinity to two main COL1A1 binding proteins: Hsp47 and fibronectin.

**Conclusions:**

Dominant inherited mutations in *COL1A1* are known causes of connective tissues disorders such as OI. These disorders are also associated with different ocular abnormalities, although recognition of the common pathology for both features is seldom being recognized. Our results expand the role of *COL1A1* mutations in different forms of early-onset glaucoma with and without signs of OI. Thus, we suggest including *COL1A1* mutation screening in the genetic work-up of glaucoma cases and detailed ophthalmic examinations with fundus analysis in patients with OI.

**Electronic supplementary material:**

The online version of this article (doi:10.1186/s13023-016-0495-y) contains supplementary material, which is available to authorized users.

## Background

Primary congenital glaucoma (PCG, OMIM 231300) is the most common type of childhood glaucoma due to the abnormal development of structures in the anterior segment of the eye including the trabecular meshwork, Schlemm’s canal and the anterior chamber angle. It manifests during neonatal or early infantile period (before 3 years of age) and is characterized by elevated intraocular pressure (IOP), increased corneal diameter, enlarged globe, Haab’s striae, corneal edema, and optic nerve head cupping. Symptoms are photophobia, ephiphora and blepharospasm [1]. PCG represents a diagnostic and therapeutic challenge as it can lead to irreversible blindness in the first years of life if untreated.

Inheritance is primarily autosomal recessive, although pedigrees with dominant inheritance or sporadic cases have been described [2]. Several chromosomal loci have been so far mapped for the recessive form of PCG, but up to now only two genes have been identified: cytochrome P4501 subfamily 1B1 (CYP1B1, OMIM 601771) on the GLC3A locus and latent transforming growth factor beta binding protein 2 (*LTBP2*, OMIM 602091) on the GLC3D locus. *CYP1B1* is the most common identifiable cause of PCG worldwide. In the European population the prevalence of *CYP1B1* mutations ranges from 20 to 30 % of all PCG cases [3]. In contrast, *LTBP2* mutations in classical cases of PCG are much rarer, being reported only for a few cases from Pakistan and in patients of Gypsy ethnicity [4].

Mutations in *CYP1B1* can infrequently underlie the autosomal dominant, juvenile open-angle glaucoma (JOAG, OMIM 137750) and even adult-onset forms of primary open-angle glaucoma (POAG, OMIM 137760) [5]. However, across most populations, the most common identifiable cause of JOAG remains heterozygous mutations in myocilin gene (*MYOC*, OMIM 601652), underlying up to 7–15 % of cases. JOAG manifests in the first decades of life and is characterized by elevated IOP, progressive glaucomatous optic neuropathy, severe visual field defects (VFD) and is frequently associated with severe myopia [6].

A further autosomal dominant form of infantile/developmental glaucoma is a phenotypic aspect of ocular anterior segment dysgenesis (ASD), a genetically heterogeneous group of complex developmental disorders including Axenfeld-Rieger’s anomaly, Peters’ anomaly, aniridia, iris hypoplasia and iridogoniodysgenesis [7]. Approximately 50 % of individuals with ASD generally develops glaucoma due to malformations of tissues responsible for the IOP regulation and aqueous humor drainage including the iris, cornea, lens, Schlemm’s canal and trabecular meshwork (TM) [7, 8]. Due to the malformations in Schlemm’s canal and TM, PCG is sometimes grouped together with the ASD disorders. In ASD glaucoma may develop during childhood (developmental glaucoma), but it is more common during adolescence or at the beginning of adulthood (early-onset glaucoma). Glaucoma associated with ASD progresses rapidly, is difficult to manage and may result in severe damage of the optic disc and visual field [9]. To date mutations in Forkhead Box C1 (*FOXC1*, OMIM 601090), Paired-Like Homeodomain transcription factor 2 (*PITX2,* OMIM 601542), and Paired Box Gene 6 (*PAX6,* OMIM 607108) genes are the most common cause of glaucoma in ASD [8]. Interestingly, mutations in *CYP1B1* are also associated with rare cases of Peters’ anomaly.

Currently, mutations in six genes, *CYP1B1*, *LTBP2*, *MYOC*, *PITX2*, *FOXC1* and *PAX6*, can explain only a fraction of all congenital/infantile/early-onset glaucoma cases worldwide suggesting the involvement of other candidates [10].

The identification of disease-causing variants in known or novel genes in children with glaucoma can have a significant impact on establishing proper diagnosis, disease risk assessment and clinical care. In fact, variable expressivity, phenotypic overlap and limited follow-up among these different early-onset glaucoma forms have often led to an incorrect or delayed definitive diagnosis. Moreover subsequent treatment may be inadequate because of advanced disease. As a consequence childhood glaucoma still causes a disproportionately high percentage of childhood blindness worldwide. Hence, an early and reliable diagnosis is essential to prevent unwanted vision loss and also to reduce the burden of childhood blindness [11].

Whole exome sequencing has been demonstrated to be highly successful in identifying disease-causing variants in rare ocular diseases [12, 13]. Thus, we decided to apply this approach to selected cases of congenital/infantile glaucoma presenting no disease-causing variants in the known associated genes.

Here, we report on the novel association of compound heterozygous variants in collagen type I alpha 1 gene (*COL1A1*, OMIM 120150) in one patient diagnosed with PCG and retinal detachment. Furthermore, three *COL1A1* heterozygous variants were identified in three patients: two with an early onset glaucoma form and one with congenital glaucoma, two of them presenting also an early-onset cataract and mild form of osteogenesis imperfecta (OI). In addition, we provide protein modeling based evidence for the pathogenicity of the variants identified.

Altogether, these findings expand the role of *COL1A1* in different forms of developmental/early onset glaucoma and show that disease-causing variants in *COL1A1* may act also as recessive alleles confirming data of previous mouse models [14]. In addition, our results support and double the number of patients in the literature with glaucoma observed in osteogenesis imperfecta (OI) [15] and serve to advise that glaucoma is also an important complication of OI.

## Methods

### Patients’ data

To identify the genetic basis of a rare form of PCG with retinal detachment, an Italian patient and his unaffected parents were selected for whole exome sequencing analysis. Additionally, to detect further disease-causing variants in *COL1A1* gene, 24 unrelated German patients diagnosed with PCG or infantile glaucoma in the first year of life [16] and two patients, one from Germany [17] and one from USA respectively, diagnosed with juvenile glaucoma and OI underwent variants screening of the *COL1A1* coding regions by Sanger sequencing.

Ophthalmological examinations included visual acuity measurements, tonometry and fundoscopy. PCG was defined by the following characteristics: (*i*) age of onset less than 3 years, (*ii*) increased corneal diameter greater than 10 mm accompanied by corneal edema and/or Haab striae and (*iii*) IOP greater than 21 mmHg and/or optic nerve cupping greater than 0.4. Any patient with other ocular abnormalities or systemic conditions, other than iris stromal hypoplasia, was excluded from the study. JOAG was defined when typical glaucomatous visual field (VF) loss on Octopus or Humphrey perimetry and glaucomatous alterations of the ONH were present with age of onset less than 40 years old and IOP greater than 21 mmHg.

### DNA extraction and Sanger sequencing analysis

Peripheral blood samples were obtained from all individuals and genomic DNA was extracted according to standard procedures (Flexi-Gene Kit (Qiagen, Germany). Complete coding region of the *CYP1B1*, *MYOC, FOXC1, PITX2, PAX6* and *COL1A1* genes including flanking intronic/UTR sequences were amplified by polymerase chain reaction (PCR) using appropriate amplification protocols. Primer sequences were selected using Primer3 software [18] and can be found in Additional file 1. Conditions used to amplify these coding regions can be provided on request. Purified PCR fragments were sequenced using Big Dye v.3.1 (Applied Biosystems, ABI, Weiterstadt, Germany) on an automated capillary sequencer, according to the manufacturer’s instructions (ABI 3730 Genetic Analyzer, Weiterstadt, Germany. Sequences were analysed using Sequencer5.1 (Gencodes) and SeqPilot (JSI medical systems) softwares. GenBank Accession Numbers NM_000104.3, NM_000261.1, NM_001453.2, NM_001204398.1, NM_001258462.1 and NM_000088.3 were used as reference sequences for *CYP1B1*, *MYOC, FOXC1, PITX2, PAX6* and *COL1A1* respectively [19], with +1 corresponding to the A of the translation initiation codon ATG in the cDNA nomenclature, according to the Human Genome Variation Society (HGVS) nomenclature guidelines.

To detect the presence of *CYP1B1, FOXC1, PITX2* and *COL1A1* exon rearrangements (deletion/duplication), multiplex ligation-dependent probe amplification (MLPA) assay (Kit-P54, P128, P219 and P271, MRC-Holland, Amsterdam, Netherlands) was used according to the manufacturer’s protocol. Raw data were analysed using MLPA-module of the Sequence Pilot software (JSI medical systems).

### Analysis of amino acid conservation

Evolutionary conservation of mutated amino acids was investigated with protein sequence alignment generated by Clustal Omega [20] and compared with data provided by UCSC Database [21].

### Whole-Exome Sequencing (WES)

**Fig. 1:**
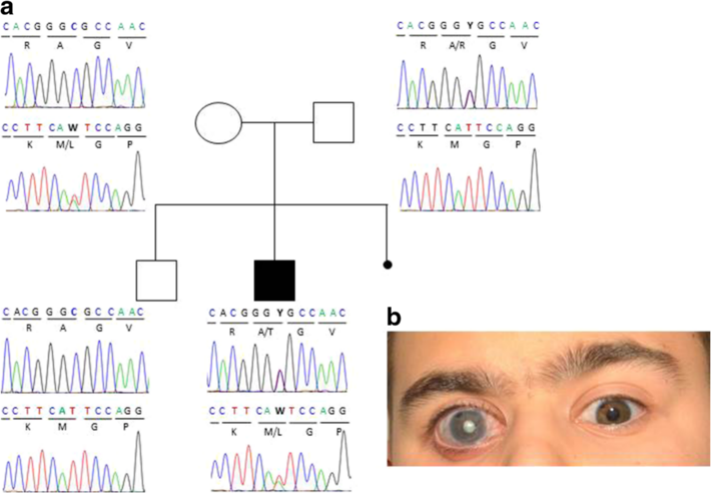
*COL1A1* mutation identified in first patient. **a** Pedigree, genotypes and DNA sequence chromatograms of affected child (*black colored symbol*), his healthy parents and brother are reported. **b** Eyes of the patient at age 15: visible are Buphthalmus mainly present in the right eye and corneal edema

For whole exome sequencing of the initial patient (ID: MI-1) and his unaffected parents, DNA samples were enriched using the SureSelect Human All Exon Kit version 5 (Agilent, Santa Clara, CA) and paired-end sequenced (75 bp forward, 25 bp reverse) on a SOLiD5500xl instrument (Life Technologies, Carlsbad, CA). Image analysis and colour calling was performed using the SOLiD instrument control software with default parameters. Read alignment to the human reference genome assembly (GRCh37/ hg19) was performed with LifeScope 2.5 using the default parameters. For the three samples, we achieved average sequence coverage of 133× (minimum 126×). On the average, 87.9 % (minimum 87.6 %) of the target sequence was covered at least 20× and 94.0 % (minimum 83.9 %) was covered at least 5×. Variants and small insertions and deletions (indels) were called using diBayes (part of LifeScope), the LifeScope small indel caller, ATLAS2-indel [22], Genome Analysis Toolkit (GATK2) and samtools/bcftools [23–25]. Variant annotation was performed using ANNOVAR [26] in conjunction with a variety of open and proprietary annotation database files: SIFT v. 1.03 [27], PolyPhen2 [28] and MutationTaster [29] were used for prediction of amino acids substitution; entries of dbSNP132 and of the 1000 Genomes Project [30] were used to check alleles frequencies. Graphical presentation of the mapped sequences was viewed with the integrative genomics viewer (IGV, [31]). A total of 86,588 variants were called, of which 3,353 were indels and 83,235 were single nucleotide variants. Based on the supposed rare incidence of the phenotype we excluded all frequent variants (above 0.01 %), detected in any of the 997 in-house control individuals of Caucasian origin as well as all annotated variants of dbSNP132, the 1000genomes project, and the exome variant server (http://evs.gs.washington.edu/EVS). Excluding intergenic, intronic and synonymous variants led to a total of seven remaining, variants in five genes proposing an autosomal recessive, X-recessive and de novo inheritance (Table 1). Based on Exome Aggregation Consortium frequencies data (ExAC, [32]) on the mutational effect of these variants, their conservation (PhyloP, GERP++) and prediction program scores of SIFT, PolyPhen2 and MutationTaster, CADD, four variants in three candidate genes remained as putative candidate variants (Table 1). Based on available knowledge annotated in different public databases as NCBI, UCSC and MGI [33–35], candidate variants in *AR* and *FMNL2* gene were excluded as these two genes could not be related to the patient phenotype. Thus, only the two variants in *COL1A1* gene remained as putative candidate variants for the given patient phenotype: one missense mutation c.790A > T (NM_000088.3: p.(Met264Leu)) in exon 11 and a second missense mutation c.3247G > A (NM_000088.3: p.(Ala1083Thr)) in exon 44 c.662C > T of the *COL1A1* gene. Both variants could be validated by Sanger sequencing and segregation was confirmed inside the family (Fig. 1). The unaffected parents are each one carrier of one of the two variants. In addition, both variants are absent in the healthy brother (Fig. 1). No further variants in any of the known glaucoma genes or other potential candidate genes were identified in this individual. Therefore, the identified variants in *COL1A1* gene are highly likely to be pathogenic.

**Table 1:** List of the exome candidate variants identified in the patient MI-1

Gene	Chrom	Position	Ref_Seq	Exon	cDNA	Protein	EVSFreq	dbSNP138	ExAC02_All	ExAC02_Eur	PhyloP	SIFT	PolyPhen2	LRT	MutationTaster	GERP++	CADD
Recessive																	
**COL1A1**	**chr17**	**48265471**	**NM_000088.3**	**44**	**c.3247G > A**	**p.(Ala1083Thr)**	**7,7E-05**	**rs372029024**	**3,277E-05**	**6,631E-05**	**1.212**	**0,09**	**P**	**D**	**D**	**4,29**	**14,23**
**COL1A1**	**chr17**	**48274385**	**NM_000088.3**	**11**	**c.790A > T**	**p.(Met264Leu)**	**7,7E-05**	**rs374947065**	**8,132E-06**	**1,477E-05**	**1.952**	**0,58**	**B**	**D**	**D**	**5,19**	**11,54**
EXPH5	chr11	108382912	NM_001144763.1	6	c.3094A > T	p.(Thr1032Ser)	0		1,627E-05	2,967E-05	0,065	0,27	B	N	N	1,75	10,15
EXPH5	chr11	108382329	NM_001144763.1	6	c.3677C > G	p.(Thr1226Arg)	0		8,132E-06	1,481E-05	0,372	0,27	B	N	N	2,91	10,69
X-Rec.																	
AR	chrX	66766013	NM_000044.2	1	c.1025C > T	p.(Pro342Leu)	0		0	0	2.317	0,18	D	N	D	4,09	14,24
IL1RAPL2	chrX	104440391	NM_017416.1	3	c.317C > T	p.(Ala106Val)	0,002461	rs144175494	0,001008	0,0017	1.081	0,48	B	N	D	3,35	14,42
De Novo																	
FMNL2	chr2	153473665	NM_052905.3	13	c.1273A > G	p.(Lys425Glu)	0		0	0	2.231	0,6	D	D	N	5,04	25,2

Main candidate variants are evidenced in bold.* Chrom* Chromosome, *EVSFreq* Exome Variant Server frequency (NHLBI Exome sequencing Project, ESP), *ExAC02* ExAC Browser (Beta), Exome Aggregation Consortium, *Eur* European

*P* probably damaging, *B* benign, *N* neutral, *D* damaging

### Molecular modelling

Mutations in collagen were modelled based on the crystal structures of isolated collagen (PDB: 3HQV [36]), collagen in complex with fibronectin (PDB: 3GXE [37]), or collagen in complex with Hsp47 (PDB: 3ZHA [38]). Amino acid exchanges were introduced with SwissModel [39]. Ligplot and Whatcheck were used for structure analysis [40, 41] and RasMol [42] was used for structure analysis and visualization [43].

## Results

### Identification of *COL1A1* variants

In this study, the first patient (ID: MI-1) is an Italian 15 year old male (Fig. 1) who was diagnosed with bilateral PCG at 4 months of age. He presented bilateral Buphthalmus, deformed ocular bulbs, corneal edema, corneal opacity, Haab’s striae, elevated IOP (40 mmHg right eye and 28 mmHg left eye, Goldmann applanation tonometry), thinning of optic nerve and ocular muscles. Following bilateral trabeculoctomy *ab externo* at the age of 6 months, the IOP was kept under control with pharmacologic therapy (Dorzolamide and Timolol). Between age 1 and 5 years he developed a high myopia (14–18 diopters, best corrected visual acuity, BCVA: 1/50 in the left and 2/10 in the right eye). At the age of six he had almost complete loss of vision with only light perception in the left eye and BCVA 1/25 in the right eye. In addition, as the proposed pharmacological therapy (Dorzolamide, Acetozulamide XR, Timolol, Travapost, Latanoprost and Brimonidine tartrate) failed in keeping the IOP under control, he underwent a further bilateral trabeculoctomy *ab externo*. Retinal detachment first in the right eye (at age 7 years) and then in the left eye (at age of 11 years) led to a complete loss of vision in both eyes despite scleral buckling and cryocoagulation surgery. Electroretinography (ERG) showed absent light response and Visual Evoked Potential (VEP) signals were completely extinct due to severe axonal damage. Both parents were healthy, without any pathological ocular phenotype.

Given the first diagnosis of PCG, mutations in *CYP1B1* and *MYOC* were excluded by conventional Sanger sequencing as well as deletions in *CYP1B1* by MLPA analysis. WES of DNA samples obtained from the patient and his unaffected parents was then carried out in order to identify novel disease causing genes. Variant analysis of patient’s and his parents’ WES data (Table 1) revealed two heterozygous candidate variants in the coding region of *COL1A1* gene, c.790A > T and c.3247G > A, which lead in the protein to the amino acid changes p.(Met264Leu) and p.(Ala1083Thr), respectively. Direct Sanger sequencing analysis on available family members demonstrated that these variants were inherited each one from one of the unaffected parents, confirming a recessive mode of inheritance. Both variants were absent in the healthy brother (Fig. 1) and could be excluded from 1.994 in house-control chromosomes.

As mutations in *COL1A1* gene are usually associated with Osteogenesis Imperfecta (OI) we performed a literature search to find possible reported cases with OI and associated glaucoma. We detected a case report on a German patient diagnosed with open angle glaucoma and a mild form of OI [17]. The described 61 year old male patient (ID: MZ-2) presented with a bilateral blue, thin and abnormally elastic sclera. Bilateral JOAG was diagnosed at the age of 29 years as reported [17]. In addition, he had an early onset cataract treated with phacoemulsification and intraocular lens (IOL)-implantation. The maximum IOP at age 54 years was 35 mmHg under medical therapy and the central cornea thickness (CCT) was extremely thin: 398 μm and 408 μm on the right and left eye, respectively. There was bilateral glaucomatous optic neuropathy and he was treated with topical application of dorzolamid, brimonidin and bimatoprost [17]. We had the opportunity to get a DNA sample from this described patient (ID: MZ-2). After exclusion of mutations in *CYP1B1* and *MYOC*, we identified by Sanger sequencing analysis of the *COL1A1* complete coding region a heterozygous variant c.757C > T. This variant introduces at protein level, a premature stop codon at amino acid position 253: p.(Arg253*). The presence of a single heterozygous variant is in accordance with an autosomal dominant inheritance model already reported both for OI and for JOAG.

**Fig. 2:**
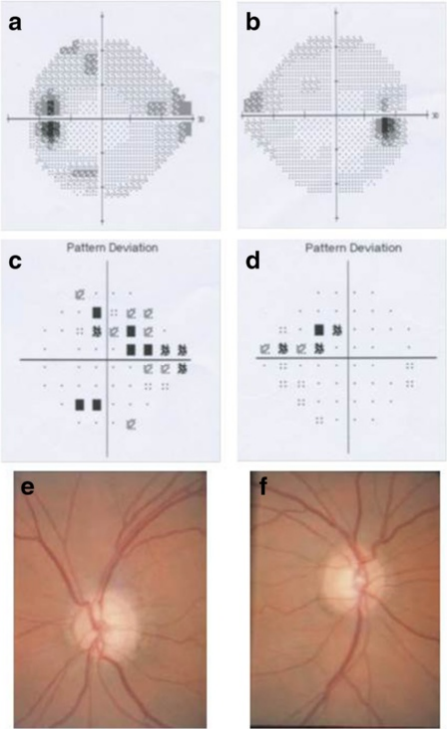
Ophthalmic findings in patient CA-3: **a**–**d** Humphrey visual fields of the third patient demonstrated early nasal field defects in both eyes (**a**/**c** left eye and **b**/**d** right eye respectively). **e**–**f** Opthic nerve photographs of patient CA-3 shown glaucomatous optic nerve cupping in both eyes (**e**-*left eye* and **f**-*right eye*)

A further third patient from USA (ID: CA-3) was referred to us for mutation screening in *COL1A1* gene. The 62 year old female had a diagnosis of OI and open angle glaucoma. The glaucoma was first diagnosed at age 55 years, although it may have developed before and not detected due to absence of detailed ophthalmic examination. In fact, the patient was under pharmaceutical therapy in both eyes (dorzolamide 2 % -timolol0.5 % bid, travaprost .003 %qhs and brimonidine 0.15 %) for elevated IOP, since 20 years. The glaucoma was reported moderately advanced, worse in left than right eye. Results of visual field exams and optic nerve photograph are shown in Fig. 2. Central corneal thickness was extremely thin (448 μm both on the right and left eye). At age 62 years, the IOP was uncontrolled under maximum tolerated medical therapy and then trabeculectomy was undertaken on the left eye. External eye exam was normal: i.e. she has no blue sclera and no corneal edema. Concerning OI diagnosis, she reported that bone changes began during adolescence and at age of 62 was wheelchair dependent. Unfortunately, we have no clinical data about the classification of OI type. As for patients ID: MZ-2, *COL1A1* Sanger sequencing analysis was performed also in this third patient (ID: CA-3) after exclusion of mutations in *MYOC* and *CYP1B1*. As expected, sequencing analysis revealed a heterozygous variant, c.2299G > A, leading at protein level to the amino acid change p.(Gly767Ser).

*COL1A1* analysis of the complete coding region by Sanger sequencing was then carried out in an independent cohort of 24 German patients diagnosed with congenital/early onset glaucoma that were found negative for mutations in *CYP1B1* and *MYOC* genes. This screening led to the identification of an additional fourth patient (ID: TU-4) harboring a heterozygous *COL1A1* variant: c.461G > T, p.(Gly154Val). Available clinical data of this 25 year old male patient are unfortunately scarce. The diagnosis of PCG was made in the first 4 years of life based on the presence of Haab’s striae. In addition, it was reported that he underwent a cataract surgery. As we do not have more detailed clinical data concerning the diagnosis of this patient we cannot exclude also to be in presence of a different form of glaucoma i.e. infantile glaucoma. Anyway, at the time of the diagnosis of PCG with cataract, clinical findings concerning OI have been not recorded.

All the parents of these last three patients (MZ-2, CA-3 and TU-4) were unavailable both for clinical reports and genetic testing, thus no familial segregation could be studied. For these three patients the sequence chromatograms of the identified variants are reported in Additional file 2.

Four of the five mutations identified (p.(Gly253*), p.(Met264Leu), p.(Ala1083Thr) and p.(Gly767Ser)) are already reported in genetic variant database dbSNP (rs72645318, rs374947065, rs372029024 and rs72651658 respectively) [44] and three (p.(Gly253*), p.(Ala1083Thr) and p.(Gly767Ser)) also in the Osteogenesis Imperfecta (OI) Variant Database [45]. The fifth mutation p.(Gly154Val) is not yet reported, both in dbSNP and OI Variant Database (Table 2). However, at position Gly154 a change into a stop codon and into alanine is already reported in both databases.

**Table 2:** COL1A1 variants identified in four glaucoma cases and related phenotype

ID	Ethnicity	Gender	Glaucoma type	Age of onset	Other ocular phenotypes	OI Diagnosis	Protein alteration	ExAC_Eur	db SNP	OI Database
MI-1	Eur	M	PCG	4 months	Retinal detachment	no	p.(Met264Leu)	6,631E-05	rs374947065	no
							p.(Ala1083Thr)	1,477E-05	rs372029024	yes
MZ-2	Eur	M	JOAG	29 years	Cataract	yes	p.(Arg253*)	0	rs72645318	yes
CA-3	Eur (Am)	F	JOAG/POAG	n/a		yes	p.(Gly767Ser)	0	rs72651658	yes
TU-4	Eur	M	PCG/Infantile glaucoma	4 years	Cataract	no	p.(Gly154Val)	0	no	no

Ethnicity: *Eur* European, *Am* American, Gender: *F* female, *M* male; Glaucoma Type: *PCG* primary congenital glaucoma, *JOAG* juvenile open angle glaucoma, *POAG* primary open angle glaucoma, *OI* osteogenesis imperfecta, *ExAC_Eur* Exome Aggregation Consortium European

Following these findings in *COL1A1,* all four present patients were additionally screened for mutations in *FOXC1, PITX2* and *PAX6*. No further variants were detected in these genes. Additionally, MLPA analysis could exclude allele deletion/insertion in *CYP1B1*, *COL1A1, FOXC1, PITX2* and *PAX6*.

### Molecular modeling

**Fig. 3:**
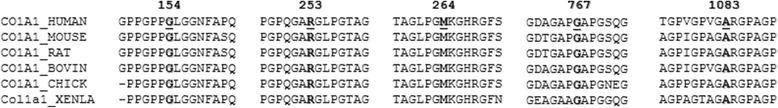
Multiple sequence alignments of human COL1A1 region to orthologous. The alignment encompasses the affected amino acid residues (in *Bold*)

**Fig. 4:**
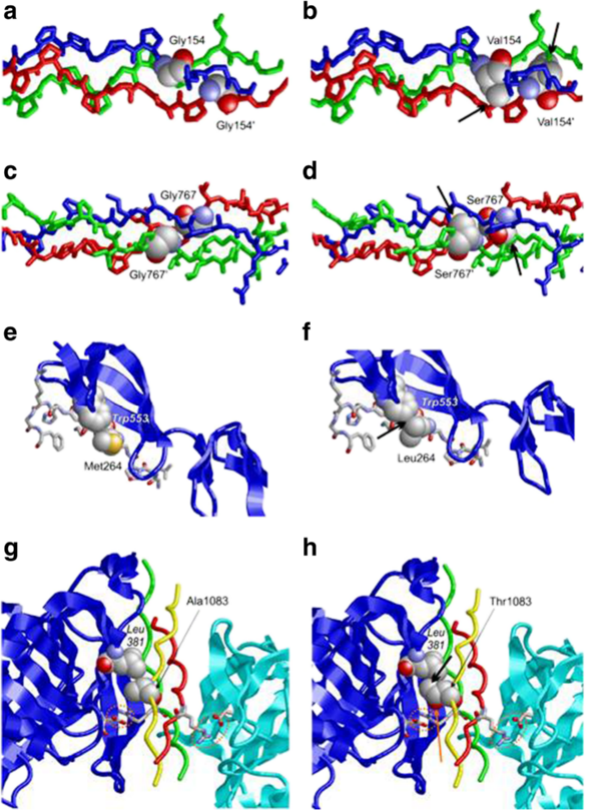
Structural effect of detected mutations in COL1A1. **a** Gly154 is located in a regular triple-helical collagen segment. The two glycines belonging to the COL1A1-chains are shown in space-filled presentation and are labelled. **b** In the Gly154Val variant, the larger valine sidechain cannot be accommodated at this sequence positions resulting in several severe clashes with the adjacent amino acids (indicated by *black arrows*). **c** Gly767 is located in a triple-helical collagen segment. The two glycines belonging to the COL1A1-chains are shown in space-filled presentation and are labelled. **d** In the Gly767Ser variant, the larger serine sidechain cannot be accommodated at this sequence positions resulting in several severe clashes with the adjacent amino acids (indicated by *black arrows*). **e** Met264 belongs to a stretch of COL1A1 that is recognized by fibronectin. Met264 forms tight sidechain packing interactions with Trp553 of fibronectin. Both residues are shown in space-filled presentation; the remaining residues of COL1A1 are shown in stick presentation (atom-type coloring) and fibronectin is shown as blue ribbon. **f** In the Met264Leu variant, the Cγ-branched leucine sidechain forms steric clashes (*black arrow*) with Trp553, which are expected to decrease binding affinity. **g** Ala1083 is located immediately adjacent to Arg1084, which confers the consensus of a high-affinity recognition site for the chaperone Hsp47 that is essential for the proper assembly of the triple-helical procollagen molecules. The collagen triple-helix is shown in red, green, and blue; the two bound Hsp47 molecules are depicted in blue and cyan. Arg1084 of collagen and Asp385 of Hsp47 form a salt-bridge that is crucial for high-affinity binding. The residues are shown in stick and their interaction is highlighted by an orange circle. Ala1083 of collagen forms a weak sidechain interaction with Leu381 of Hsp47 (residues in space-filled presentation). **h** In the Ala1083Thr variant, the bulkier threonine sidechain causes steric clashes with Leu381 (*black arrow*). Further, Ala1083 sidechain hydroxyl group is positioned close to Arg1084 (*orange arrow*), which might interfere with the Arg1084-Asp385 hydrogen bond. Both effects are expected to decrease the collagen-Hsp47 interaction

All affected amino acid positions identified are evolutionary conserved among orthologues indicating severe functional restriction at these positions (Fig. 3). Molecular modeling predicts that the three missense mutations affect either residues relevant for the collagen alpha-1(I) chain hetero-trimer stabilization (Gly154, Gly767) or residues relevant for collagen protein-protein interactions (Met264 and Ala1083) (Fig. 4).

The amino acids glycine (Gly) 154 and 767 are located in regular triple-helical segments of collagen alpha-1(I) chain (Fig. 4a, c). Gly positions are strictly conserved in the collagen proteins because it is the only amino acid lacking a side chain thus allowing the narrow association of collagen fibers within the heterotrimeric complex, facilitating inter strand hydrogen bonding. In the p.(Gly154Val) mutant, the larger side chain of valine (Val) cannot be accommodated at this sequence position resulting in severe clashes with adjacent amino acids (Fig. 4b). The same effect is also observed for the p.(Gly767Ser) mutant (Fig. 4d). Both amino acids exchanges are therefore predicted to impair the correct folding of the collagen triple-helix, a well-known pathomechanism in OI.

Amino acid methionine (Met) at position 264 is located within the fibronectin binding site of the collagen alpha-1(I) chain protein [37]. At this position Met 264 forms tight sidechain packing interactions with the tryptophan (Trp) 553 of fibronectin (Fig. 4e). These interactions are lost when Met 264 is substituted by the Cγ-branched sidechain amino acid leucine (Leu) which forms steric clashes with Trp553. These clashes are thus predicted to decrease the binding affinity of the collagen alpha-1(I) chain protein with fibronectin (Fig. 4f). The amino acid alanine (Ala) at position 1083, located immediately adjacent to arginine (Arg) 1084, is part of a high-affinity recognition consensus site for protein chaperone Hsp47. Arg1084 in collagen protein forms a salt-bridge with the aspartic acid (Asp) at position 385 of Hsp47 protein (Fig. 4g) which is crucial for the binding between the two proteins. Substitution of Ala1083 by threonine at position 1083 has two effects due its bulkier sidechain containing a hydroxyl-group. The first effect is to cause some steric clashes with Leu381 and the second to interfere with the hydrogen bond formed by amino acids Arg1084-Asp385 (Fig. 4h). Both effects are expected to decrease the interaction of the collagen alpha-1(I) chain with Hsp47.

## Discussion

The collagen alpha-1(I) chain protein belongs to the collagen complex superfamily in which each component has a specific function, or set of functions, and there are extensive interactions with other connective tissue components [46]. Thus the clinical phenotypes resulting from collagen mutations are wide-ranging in their manifestations and severity [47]. Of the 28 known vertebrate collagen types, type I collagen is the most abundant and widely expressed collagen in humans. It is a heterotrimer comprising two alpha 1 (I) chains and one alpha 2 (I) chain. The alpha 1 (I) and alpha 2 (I) chains of type I collagen are encoded at the unlinked loci *COL1A1* and *COL1A2,* respectively [48]. The most striking feature of these alpha chains is that they consist of repeating Gly-Xaa-Yaa tri-peptides motifs. The presence of glycine every third amino acid is essential to allow the alpha chain to adopt the characteristic collagen triple helix. The mutation profiles of these genes are not restricted to any specific region but are scattered throughout the entire structural domains and show enormous diversity. So far, more than 800 mutations have been reported only for *COL1A1* [45], mainly associated with skeletal and dermatological conditions such as OI, Ehlers-Danlos syndrome (EDS), bone mineral density variation, osteoporosis and Caffey disease. However, in the literature there are different descriptions in which both OI and EDS patients present also a variety of ocular abnormalities. Characteristic ocular findings described are usually blue sclera, thin cornea, microcornea, myopia, keratoconus, congenital absence of Bowman’s layer, retinal detachment, glaucoma [49–51]. This is not surprising since up to 80 % of eye tissues are composed of different collagen proteins, and the most abundant is the type I collagen, in particular in cornea, sclera, iris, ciliary body, trabecular meshwork and optic nerve [52]. Additionally, genome wide association studies (GWAS) have shown that variants at collagen-related genes, including *COL1A1*, influence one of the main glaucoma risk factor such as central corneal thickness (CCT) [53, 54].

Mutations in certain collagen genes have also been associated with glaucoma manifesting as part of a systemic disorder. Stickler syndrome (STL, OMIM 108300), for example, is a group of diseases primarily caused by mutations in the fibril-forming collagen type II gene. Mutations causing premature stop codons in exon 2 of *COL2A1* lead to ocular-only phenotypes including retinal detachment and high myopia, but with few or no other systemic manifestations [55]. A recent GWAS study found significant association of a single nucleotide polymorphism in *COL11A1* with primary angle-closure glaucoma [56]. Further, genetic variants in collagen XV, alpha 1 (*COL15A1*) and collagen XVIII, alpha 1 (*COL18A1*) have been shown to modify the age of onset of both JOAG and POAG [57].

Collagen proteins are also a pivotal component of the extracellular matrix (ECM) of the TM, Schlemm’s canal (SC) and lamina cribrosa (LC), which represent the ocular tissues involved in glaucoma development. Morphological and ultrastructural ECM changes involving elastic-fibers and microfibrils have been already implicated in glaucoma pathogenesis [58]. In addition, mutations in multiple genes encoding elastic microfibril components have been linked to glaucoma [59]. For example, fibrillin-1 (*FBN1*) mutations cause Marfan syndrome (OMIM 154700) and ocular abnormalities including ectopia lentis, myopia and glaucoma [49, 60]. Mutations in *LTBP2*, which binds to fibrillin-containing microfibrils, cause not only PCG, but also POAG and pseudoexfoliation glaucoma [61, 62]. Altogether, these observations underline the importance of collagen genes, including *COL1A1*, in the eye and their putative role in glaucoma pathogenesis. Nevertheless, except for a recent report by Wallace et al., [15] there is no detailed documentation about the association of *COL1A1* mutations and glaucoma with and/ or without sign of OI and EDS.

In the present study we describe *COL1A1* variants in four patients associated with different glaucoma forms: two presenting with PCG, two with JOAG and/or an early form of POAG. Both patients with early form of open angle glaucoma present also a diagnosis of OI: in particular a mild OI type I in the MZ-2 patient, while the third patient CA-3 has a more severe but not classified OI form (Table 2).

Of particular interest is the novel finding of *COL1A1* compound heterozygous variants (p.(Met264Leu) and p.(Ala1083Thr)) in the patient MI-1 presenting a novel phenotype of PCG, retinal detachment and light ligaments laxity (Table 2). Molecular modelling suggests that these two amino acid changes impair the collagen protein complex interaction with two different binding proteins: Hsp47 and fibronectin. Thus, the mechanism of these two variants is different from the one of the most frequently reported missense variants causing mainly OI and EDS that is usually dominant and affects the formation and stabilization of the collagen alpha-1(I) chain triple-helix itself similarly to the case of our three other patients described (Table 2). Instead, in the recessive mode the collagen alpha-1(I) chain protein interactions with binding partners seem to be affected. Hsp47 is important for the proper assembly of the triple-helical procollagen molecules and has an important role in eye morphogenesis [38]. Fibronectin has been found to be expressed in TM, ciliary body, choroid, basement membrane of the corneal epithelium, corneal stroma and Descemet’s membrane. It has been shown to be essential in cornea morphogenesis and in stabilizing the vitreoretinal attachment [63]. Impaired binding of collagen protein to Hsp47 and fibronectin could thus affect the correct formation of eye tissues important for outflow, IOP regulation and retinal attachment. Thus our results suggest that concomitant impairment of both copies of COL1A1 with respective binding proteins could influence at a very early stage the development of the eye more than other tissues. This might explain the more severe ocular phenotype observed in patient MI-1 compared to the other patients carrying variants with dominant effect.

Although, so far *COL1A1* gene mutations in humans are overwhelmingly dominant in their action, there is a single example of a recessively-inherited case of OI caused by a homozygous missense mutation in *COL1A2* gene [64]. Remarkably, three studies on transgenic mice with targeted missense mutations in *COL1A1* (not-affecting positions of amino acid Gly in the triple-helices) revealed a glaucoma-like phenotype supporting the causality of the variants identified in our patient. The mice developed sustained elevation of IOP, progressive optic nerve damage and showed a reduced outflow facility. In addition, they do not present typical clinical signs of OI and EDS like in our first patient. Further, the heterozygous mice carriers have been reported to be without any signs of OI and glaucoma similar phenotype as the parents of our MI-1 patient analyzed [14, 65, 66].

The p.(Arg253*) identified in patient MZ-2 introduces a premature stop codon in the *COL1A1* gene coding sequence. This mutation has been previously reported in a 13 year old patient diagnosed with a mild form of OI and blue sclera [67]. Glaucoma was not described for that patient maybe due to the earlier age (13 years) compared to the onset age of 29 years in our MZ-2 patient with JOAG and mild OI form. A large number of frameshifts/nonsense mutations in this gene have been reported to be associated with the mild OI type I, following classical dominant inheritance. Frameshift and nonsense sequence variants usually result in nonsense mediated decay (NMD) of affected transcripts leading to a reduced levels of mRNA. In practice, this means that little or no truncated protein product is formed resulting in the production of about half the amount of normal type I collagen [68]. Decreased amount of collagen alpha-1(I) chain protein in the eye could thus explain the thin and abnormal sclera and the glaucoma detected in our patient MZ-2.

The mutation identified in patient CA-3 and TU-4 removes a glycine (Gly) at position 154 and 767 introducing a valine (Val) and serine (Ser) respectively. Mutations affecting glycine position which produce an abnormal collagen protein are usually reported in more severe phenotypes than nonsense do. This could explain the presence of a severe OI form in the patient CA-3 in which bone deformities started already in adolescence and the presence of a congenital glaucoma in the fourth patient identified. Clinical signs of OI or EDS for this last patient, TU-4, were not yet reported maybe due to the early age of the patient’s glaucoma diagnosis (4 years).

We suggest that the range of *COL1A1* mutations presenting as either dominant or recessive differentially affect the collagen alpha-1(I) chain protein function resulting in a spectrum of connective tissue related phenotypes of varying severity including glaucoma features as recently proposed [15]. Nevertheless, we cannot exclude the possible presence of unidentified genetic variants located outside the regions covered by WES, acting in concert with *COL1A1* to modulate the phenotype.

One of the possible limitations of this study due to the rareness of these combined phenotypes could be the small number of affected people screened. A further limitation might be also the absence of unaffected relatives in the three patients, carriers of single heterozygous variants, to prove the dominant inheritance and penetrance of these variants. However, taking into account that these variants are extremely rare (Table 2) and that they show a putative impairing effect on the protein function (Fig. 4), we can suggest that these are probably pathogenic. Undoubtedly, it is necessary widening the screening of *COL1A1* in different, larger cohort of patients with glaucoma and additionally also OI to replicate and confirm these findings.

## Conclusions

In summary, the present data together with the recent literature support and expand the role of *COL1A1* in the eye pathology suggesting a putative role in different forms of glaucoma. Screening of *COL1A1* in larger cohorts of patients with different forms of glaucoma would be now necessary to establish a detailed phenotype-genotype correlation. On the other side, also patients with different forms of OI and EDS should be regularly subjected to ophthalmologic examination lifelong not only to prevent irreversible ocular damages but also to have a correct estimate about glaucoma prevalence in these disorders.

## Abbreviations

ASD, anterior segment dysgenesis; BCVA, best correct visual acuity; CYP1B1, cytochrome P4501 subfamily 1B1; COL1A1, collagen type I alpha 1 gene; EDS, Ehlers-Danlos syndrome; FOXC1, forkhead Box C1 gene; Hsp47, heat-shock protein 47; IOP, intraocular pressure; GLC3A, GLC3D etc. primary congenital glaucoma locus A, D; JOAG, juvenile open angle glaucoma; LTBP2, latent transforming growth factor beta binding protein 2; MYOC, myocillin gene; MLPA, multiplex ligation-dependent probe amplification; OI, osteogenesis imperfecta; PAX6, Paired Box Gene 6; PCG, primary congenital glaucoma; PCR, polymerase chain reaction; PDB, protein database; POAG, primary open angle glaucoma; PITX2, paired-like homeodomain transcription factor 2; WES, whole exome sequencing

## Additional files

Additional file 1: PCR Primers used for molecular screening of *CYP1B1, MYOC, FOXC1, PITX2, PAX6* and *COL1A1* genes. (DOCX 20 kb) Additional file 2: Sequence chromatograms of the variants identified in *COL1A1* gene in three different patients: MZ-2, CA-3 and TU-4. (DOCX 129 kb)

## References

[1] Walton DS (1979). Primary congenital open angle glaucoma: a study of the anterior segment abnormalities. Trans Am Ophthalmol Soc.

[2] Francois J (1980). Congenital glaucoma and its inheritance. Ophthalmologica.

[3] Stoilov I, Akarsu AN, Sarfarazi M (1997). Identification of three different truncating mutations in cytochrome P4501B1 (CYP1B1) as the principal cause of primary congenital glaucoma (Buphthalmos) in families linked to the GLC3A locus on chromosome 2p21. Hum Mol Genet.

[4] Ali M, McKibbin M, Booth A, Parry DA, Jain P, Riazuddin SA, Hejtmancik JF, Khan SN, Firasat S, Shires M (2009). Null mutations in LTBP2 cause primary congenital glaucoma. Am J Hum Genet.

[5] Pasutto F, Chavarria-Soley G, Mardin CY, Michels-Rautenstrauss K, Ingelman-Sundberg M, Fernandez-Martinez L, Weber BH, Rautenstrauss B, Reis A (2010). Heterozygous loss-of-function variants in CYP1B1 predispose to primary open-angle glaucoma. Invest Ophthalmol Vis Sci.

[6] Lotufo D, Ritch R, Szmyd L, Burris JE (1989). Juvenile glaucoma, race, and refraction. JAMA.

[7] Ito YA, Walter MA (2014). Genomics and anterior segment dysgenesis: a review. Clin Experiment Ophthalmol.

[8] Gould DB, John SW (2002). Anterior segment dysgenesis and the developmental glaucomas are complex traits. Hum Mol Genet.

[9] Strungaru MH, Dinu I, Walter MA (2007). Genotype-phenotype correlations in Axenfeld-Rieger malformation and glaucoma patients with FOXC1 and PITX2 mutations. Invest Ophthalmol Vis Sci.

[10] Fan BJ, Wiggs JL (2010). Glaucoma: genes, phenotypes, and new directions for therapy. J Clin Invest.

[11] Chen TC, Chen PP, Francis BA, Junk AK, Smith SD, Singh K, Lin SC (2014). Pediatric glaucoma surgery: a report by the American Academy Of Ophthalmology. Ophthalmology.

[12] Gonzalez-del Pozo M, Mendez-Vidal C, Bravo-Gil N, Vela-Boza A, Dopazo J, Borrego S, Antinolo G (2014). Exome sequencing reveals novel and recurrent mutations with clinical significance in inherited retinal dystrophies. PLoS One.

[13] Prokudin I, Simons C, Grigg JR, Storen R, Kumar V, Phua ZY, Smith J, Flaherty M, Davila S, Jamieson RV (2014). Exome sequencing in developmental eye disease leads to identification of causal variants in GJA8, CRYGC, PAX6 and CYP1B1. Eur J Hum Genet.

[14] Mabuchi F, Lindsey JD, Aihara M, Mackey MR, Weinreb RN (2004). Optic nerve damage in mice with a targeted type I collagen mutation. Invest Ophthalmol Vis Sci.

[15] Wallace DJ, Chau FY, Santiago-Turla C, Hauser M, Challa P, Lee PP, Herndon LW, Allingham RR (2014). Osteogenesis imperfecta and primary open angle glaucoma: genotypic analysis of a new phenotypic association. Mol Vis.

[16] Weisschuh N, Wolf C, Wissinger B, Gramer E (2009). A clinical and molecular genetic study of German patients with primary congenital glaucoma. Am J Ophthalmol.

[17] Rosbach J, Vossmerbaeumer U, Renieri G, Pfeiffer N, Thieme H (2012). Osteogenesis imperfecta and glaucoma. A case report. Ophthalmologe.

[18] Primer3 Design (v.0.4.0). [http://bioinfo.ut.ee/primer3-0.4.0/]

[19] National Centre for Biotechnology Information. [http://www.ncbi.nlm.nih.gov/]

[20] Multiple Sequence Alignment. [http://www.ebi.ac.uk/Tools/msa/]

[21] UCSC Genome Browser. [http://genome.ucsc.edu/]

[22] Challis D, Yu J, Evani US, Jackson AR, Paithankar S, Coarfa C, Milosavljevic A, Gibbs RA, Yu F (2012). An integrative variant analysis suite for whole exome next-generation sequencing data. BMC Bioinformatics.

[23] Li H, Durbin R (2009). Fast and accurate short read alignment with Burrows-Wheeler transform. Bioinformatics.

[24] Li H, Handsaker B, Wysoker A, Fennell T, Ruan J, Homer N, Marth G, Abecasis G, Durbin R, Genome Project Data Processing S (2009). The Sequence Alignment/Map format and SAMtools. Bioinformatics.

[25] McKenna A, Hanna M, Banks E, Sivachenko A, Cibulskis K, Kernytsky A, Garimella K, Altshuler D, Gabriel S, Daly M (2010). The genome analysis toolkit: a MapReduce framework for analyzing next-generation DNA sequencing data. Genome Res.

[26] Wang K, Li M, Hakonarson H (2010). ANNOVAR: functional annotation of genetic variants from high-throughput sequencing data. Nucleic Acids Res.

[27] SIFT Prediction. [http://sift.jcvi.org/]

[28] PolyPhen2 (Polymorphism Phenotyping v2). [http://genetics.bwh.harvard.edu/pph2/]

[29] Mutation Taster. [http://www.mutationtaster.org/]

[30] The 1000 Genomes Project (Phase 1). [http://www.1000genomes.org]

[31] Integrative Genomics Viewer. [http://www.broadinstitute.org/igv/]

[32] Exome Aggregation Consortium (ExAC). [http://exac.broadinstitute.org]

[33] National Center for Biotechnology Information. [http://www.ncbi.nlm.nih.gov/]

[34] UC Santa Cruz Genome Bioinformatics. [http://genome.ucsc.edu/]

[35] Mouse Genome Informatics. [http://www.informatics.jax.org/]

[36] Orgel JP, Irving TC, Miller A, Wess TJ (2006). Microfibrillar structure of type I collagen in situ. Proc Natl Acad Sci U S A.

[37] Erat MC, Sladek B, Campbell ID, Vakonakis I (2013). Structural analysis of collagen type I interactions with human fibronectin reveals a cooperative binding mode. J Biol Chem.

[38] Widmer C, Gebauer JM, Brunstein E, Rosenbaum S, Zaucke F, Drogemuller C, Leeb T, Baumann U (2012). Molecular basis for the action of the collagen-specific chaperone Hsp47/SERPINH1 and its structure-specific client recognition. Proc Natl Acad Sci U S A.

[39] Guex N, Peitsch MC (1997). SWISS-MODEL and the Swiss-PdbViewer: an environment for comparative protein modeling. Electrophoresis.

[40] LigPlot + v.1.4-multiple ligand-protein interaction diagrams for drug discovery. [http://www.ebi.ac.uk/thornton-srv/software/LigPlus/] 10.1021/ci200227u 21919503

[41] What_Check. [http://swift.cmbi.ru.nl/gv/whatcheck/]

[42] RasMol and OpenRasMol: Molecular Graphics Visualization Tool. [http://www.openrasmol.org/]

[43] Hooft RW, Vriend G, Sander C, Abola EE (1996). Errors in protein structures. Nature.

[44] Database of single nucleotide polymorphisms (SNPs). [http://www.ncbi.nlm.nih.gov/snp/]

[45] Osteogenesis Imperfecta Variant Database. [https://oi.gene.le.ac.uk]

[46] Brown JC, Timpl R (1995). The collagen superfamily. Int Arch Allergy Immunol.

[47] Prockop DJ, Kivirikko KI (1995). Collagens: molecular biology, diseases, and potentials for therapy. Annu Rev Biochem.

[48] Dalgleish R (1997). The human type I collagen mutation database. Nucleic Acids Res.

[49] Chan CC, Green WR, de la Cruz ZC, Hillis A (1982). Ocular findings in osteogenesis imperfecta congenita. Arch Ophthalmol.

[50] Madigan WP, Wertz D, Cockerham GC, Thach AB (1994). Retinal detachment in osteogenesis imperfecta. J Pediatr Ophthalmol Strabismus.

[51] Nwosu BU, Raygada M, Tsilou ET, Rennert OM, Stratakis CA (2005). Rieger's anomaly and other ocular abnormalities in association with osteogenesis imperfecta and a COL1A1 mutation. Ophthalmic Genet.

[52] Mietz H, Kasner L, Green WR (1997). Histopathologic and electron-microscopic features of corneal and scleral collagen fibers in osteogenesis imperfecta type III. Graefes Arch Clin Exp Ophthalmol.

[53] Dimasi DP, Chen JY, Hewitt AW, Klebe S, Davey R, Stirling J, Thompson E, Forbes R, Tan TY, Savarirayan R (2010). Novel quantitative trait loci for central corneal thickness identified by candidate gene analysis of osteogenesis imperfecta genes. Hum Genet.

[54] Vithana EN, Aung T, Khor CC, Cornes BK, Tay WT, Sim X, Lavanya R, Wu R, Zheng Y, Hibberd ML (2011). Collagen-related genes influence the glaucoma risk factor, central corneal thickness. Hum Mol Genet.

[55] Tran-Viet KN, Soler V, Quiette V, Powell C, Yanovitch T, Metlapally R, Luo X, Katsanis N, Nading E, Young TL (2013). Mutation in collagen II alpha 1 isoforms delineates Stickler and Wagner syndrome phenotypes. Mol Vis.

[56] Vithana EN, Khor CC, Qiao C, Nongpiur ME, George R, Chen LJ, Do T, Abu-Amero K, Huang CK, Low S (2012). Genome-wide association analyses identify three new susceptibility loci for primary angle closure glaucoma. Nat Genet.

[57] Wiggs JL, Howell GR, Linkroum K, Abdrabou W, Hodges E, Braine CE, Pasquale LR, Hannon GJ, Haines JL, John SW (2013). Variations in COL15A1 and COL18A1 influence age of onset of primary open angle glaucoma. Clin Genet.

[58] Tektas OY, Lutjen-Drecoll E (2009). Structural changes of the trabecular meshwork in different kinds of glaucoma. Exp Eye Res.

[59] Kuchtey J, Kuchtey RW (2014). The microfibril hypothesis of glaucoma: implications for treatment of elevated intraocular pressure. J Ocul Pharmacol Ther.

[60] Izquierdo NJ, Traboulsi EI, Enger C, Maumenee IH (1992). Glaucoma in the Marfan syndrome. Trans Am Ophthalmol Soc.

[61] Desir J, Sznajer Y, Depasse F, Roulez F, Schrooyen M, Meire F, Abramowicz M (2010). LTBP2 null mutations in an autosomal recessive ocular syndrome with megalocornea, spherophakia, and secondary glaucoma. Eur J Hum Genet.

[62] Jelodari-Mamaghani S, Haji-Seyed-Javadi R, Suri F, Nilforushan N, Yazdani S, Kamyab K, Elahi E (2013). Contribution of the latent transforming growth factor-beta binding protein 2 gene to etiology of primary open angle glaucoma and pseudoexfoliation syndrome. Mol Vis.

[63] Kohno T, Sorgente N, Ishibashi T, Goodnight R, Ryan SJ (1987). Immunofluorescent studies of fibronectin and laminin in the human eye. Invest Ophthalmol Vis Sci.

[64] Pihlajaniemi T, Dickson LA, Pope FM, Korhonen VR, Nicholls A, Prockop DJ, Myers JC (1984). Osteogenesis imperfecta: cloning of a pro-alpha 2(I) collagen gene with a frameshift mutation. J Biol Chem.

[65] Aihara M, Lindsey JD, Weinreb RN (2003). Ocular hypertension in mice with a targeted type I collagen mutation. Invest Ophthalmol Vis Sci.

[66] Dai Y, Lindsey JD, Duong-Polk X, Nguyen D, Hofer A, Weinreb RN (2009). Outflow facility in mice with a targeted type I collagen mutation. Invest Ophthalmol Vis Sci.

[67] Venturi G, Tedeschi E, Mottes M, Valli M, Camilot M, Viglio S, Antoniazzi F, Tato L (2006). Osteogenesis imperfecta: clinical, biochemical and molecular findings. Clin Genet.

[68] Willing MC, Deschenes SP, Slayton RL, Roberts EJ (1996). Premature chain termination is a unifying mechanism for COL1A1 null alleles in osteogenesis imperfecta type I cell strains. Am J Hum Genet.

