# Supplementary material for: Expanding the clinical spectrum of COL1A1 mutations in different forms of glaucoma
Source: Orphanet J Rare Dis. 2016 Aug 2;11:108. doi: 10.1186/s13023-016-0495-y (PMC4970237; doi:10.1186/s13023-016-0495-y)
Supplement: Additional file 1: — PCR Primers used for molecular screening of CYP1B1, MYOC, FOXC1, PITX2, PAX6 and COL1A1 genes. (DOCX 20 kb) [file 13023_2016_495_MOESM1_ESM.docx]

**Additional file 1**

PCR Primers used for molecular screening of CYP1B1, MYOC, FOXC1, PITX2, PAX6 and COL1A1 genes

| Exon-fragments | Forward primer (5'-3') | Reverse primer (5'-3') |
| --- | --- | --- |
| CYP1B1_e2p1 | ACCCAACGGCACTCAGTC | CGAGTAGTGGCCGAAAGC |
| CYP1B1_e2p2 | ATAGTGGTGCTGAATGGCG | GGAAGTACTGCAGCCAGGG |
| CYP1B1_e2p3 | CTACAGCCACGACGACCC | GCATATTCTGTCTCTACTCCGC |
| CYP1B1_e3p1 | TTTTGCTCACTTGCTTTTCTCT | TAGAAAGTTCTTCGCCAATGC |
| CYP1B1_e3p2 | GCCTGTCACTATTCCTCATGC | CAGCTTGCCTCTTGCTTCTTA |
| CYP1B1_e3p3 | TGTGAATCATGACCCAGTGAA | TTCATTGGGCCCTTTAAGTCT |
|  |  |  |
| MYOC_ex1ASF | CTCTGTCTTCCCCCATGAAG | CTGGTCCAAGGTCAATTGGT |
| MYOC_ex1BS | AGGCCATGTCAGTCATCCATA | AGCAGGTCACTACGAGCCATA |
| MYOC_ex2 | ATAGTCAATCCTTGGGCCATT | TCTGCTCCCAGGGAAGTTAAT |
| MYOC_ex3AS | CTCCAGGGCTGTCACATCTAC | ATCCACAGCCAAGTCAATGTC |
| MYOC_ex3BS | CTACCCCTACACCCAGGAGAC | TGGTCAGGGTCTTGCTGATAC |
| MYOC_ex3CS | GACATTGACTTGGCTGTGTGGAT | GACCATGTTCATCCTTCTGGA |
|  |  |  |
| FOXC1-EX1 | CCCGGACTCGGACTCG | AGATGCCGTTCAGGGTGATC |
| FOXC1-EX2 | GACATGGTGAAGCCGCCCTAT | CACGTACCGTTCTCGGTCTT |
| FOXC1-EX3 | AAGGAGGAGAAGGACAGGCT | AGCTGTAGAGGGAGCTCTGG |
| FOXC1-EX4 | TTCAGCGTGGACAACATCAT | TCAGGTACCACGAGGTGAGG |
| FOXC1-EX5 | TACTCTCTGCCTCCGGTCAC | TATTCTGTTCGCTGGTGTGG |
| FOXC1-EX6 | TACTCTCTGCCTCCGGTCAC | TTAGTTTCGATTTTGCCTTG |
|  |  |  |
| PITX2-EX4 | AGTCTCATCTGAGCCCTGCT | CTGGCGATTTGGTTCTGATT |
| PITX2-EX5 | TTTGCTCTTTGTCCCTCTTTC | CTCGCCTTCTTGCGCTTC |
| PITX2-EX6 | CAGCTCTTCCACGGCTTCT | GGCCTGTACCTCCACAACAT |
| PITX2-EX7p1 | TTAGTAATCTGCACTGTGGC | CCACTGCATACTGGCAAGCA |
| PITX2-EX7p2 | GAGTAGCCCGTCGCTGAAT | TGTGTATGTGTCCTTGCAACTG |
|  |  |  |
| PEX6-EX4 | AAGCCCCAAAGGGTAGATTTT | TATCGAGAAGAGCCAAGCAAA |
| PAX6-EX5 | TTGTCCTTTATTTGATCGATAGCA | GGGTCCATAATTAGCATCGTTTAC |
| PAX6-EX6 | CACTTTAAGCAAGGTCAGCACA | TCGCTACTCTCGGTTTACTACCA |
| PAX6-EX7 | AAAGTCCAAGTGCTGGACAATC | AGGTAAAGAGGAGAGAGCATTGG |
| PAX6-EX8 | GAGATGGGTGACTGTGTCTTCA | AGAGGAAATGGTTGGGAGAGTAG |
| PAX6-EX9 | AAGAAGGCTGACAGTTACCTTGG | CAAAGGGCCCTGGCTAAAT |
| PAX6-EX10 | GTGGGAAAGTTCTTCCAAGTACAG | CAGAGCATTTAGCAGACTGAACC |
| PAX6-EX11 | TTTCCTAGAGACAGAGGTGCTTG | CAGATGTGAAGGAGGAAACTGAG |
| PAX6-EX12 | CAGTGTCTACCAACCAATTCCAC | GATTGACTGTCTCCGACTTGACT |
| PAX6-EX13 | CAGACTTGTTGGCAGAGTTCC | GGACAAGGAAAGCAAGGAGTT |
| PAX6-EX14 | GCTCCTCTAGACCTTTTGCTG | AAGTCCATTCCTTCCCCAGT |
|  |  |  |
| Exon-fragments | Forward primer (5'-3') | Reverse primer (5'-3') |
| COL1A1-E1 | GGGCAGGGTTCCTCCCTGCT | AGGAAGAGCCCTCATCATCTC |
| COL1A1-E2-3 | TCCAAGTGTGCCTCTTAGACC | AGAAGGGAGGACTGTGAGGAG |
| COL1A1-E4-5 | TCTCCTGCCCTCGAATTTTGC | GCACATGTCACAAACTGTGAAG |
| COL1A1-E6-7 | TTCAATTCACTGTCCTCACTC | CTTCCCTCCAAAAGACCAAAG |
| COL1A1-E8-9 | GTCTTTTGGAGGGAAGACTGG | CCCTCCTTCCTCTGAGTATCG |
| COL1A1-E10-11 | GACCTGCAACAATCCAAAGAA | CCCTTGGGACTTCTGTAGCT |
| COL1A1-E12-13 | TCCCAAGGCTCTTTCTCAGAT | GATGGGAGACAGCCTTGTTCC |
| COL1A1-E14-15 | GAACAAGGCTGTCTCCCATCT | CTGGGGTTCAGACCAACATAA |
| COL1A1-E16 | AGGTTATGTTGGTCTGAACCC | AGGGACAGGAGAGCAATGATC |
| COL1A1-E17-18 | CTGATCATTGCTCTCCTGTCC | TGTGGGAGGCAGACAGCCAG |
| COL1A1-E19-20 | GGCTTCAGTCCCACTCCTG | GACTAGGGGCTCCTCTTCCT |
| COL1A1-E21-22 | AGGAACCCCTGACACTGGA | CTCAAGTTTGTGGCTCTTTGC |
| COL1A1-E23 | CTGGGCATCTTCTTCCTCTTT | GCCTCATCCCAGACCCTACA |
| COL1A1-E24-25 | TGTAGGGTCTGGGATGAGGC | GCTGAGGTCCAGAAAGTGAGA |
| COL1A1-E26-27 | TCCCTTAAAAAGTCCCACTCA | TTTCCTACCCCTACCTCCCA |
| COL1A1-E28-29 | GAGATCTGGGGAGCAGAAAAG | TGGCTGTCTGATTAGCTAGGAG |
| COL1A1-E30-31 | AGGGGTTCCTCTCTAATCACG | ACACCCTATCTCCATGGCTTT |
| COL1A1-E32 | GGAAACAAGCCTGGGAGATAC | AGAGGGACAGATCCCAGAGAG |
| COL1A1-E33 | AAACCCAGACACAAGCAGAAC | AGTAGATGACCCCAGGAGAGC |
| COL1A1-E34-35 | GCTCTCCTGGGGTCATCTACT | AGTAATGGAGGCAGGAAGATG |
| COL1A1-E36-37 | CTTCCTGCCTCCATTACTGCT | AGTAGCACCCTGGAAGGAGAG |
| COl1A1-E38 | TGGCAGGTGAGGGCAGCTGGG | TCATTGGGTCCTCAGTCAGCC |
| COL1A1-E39-40 | GGCTGACTGAGGACCCAATGA | CCAAGTCCTGTGATGGTTTTT |
| COL1A1-E41-42 | AAAACCATCACAGGACTTGGAGTG | CAGGGAAGCAGCAGACAAG |
| COL1A1-E43 | GCAACACTCCATGACCACAGC | TTTTTGGATTAAGGCCCTGAC |
| COL1A1-E44-45 | TGAGGAGAGAGAGATCCAGCA | CACTAGGGAGGGGAAAGAATG |
| COL1A1-E46 | CTCGGGCTAGATTTCCAGAAT | AGCAGAGAGGCCAAAGCTAGA |
| COL1A1-E47 | GCTATGGCATGTCCTATGGA | TGGGGATTACCGGCATCCA |
| COL1A1-E48 | TGGATGCCGGTAATCCCCAC | CCAGTACTCTCCTGTGGTAGGG |
| COL1A1-E49 | CTAGCCTCTCCCTCCCTCCTA | CTTCATGTCCCTTCTGAGCAC |
| COL1A1-E50-51 | TATAACGGTGCATTGGGCAGC | GTTTGGGTTGCTTGTCTGTTT |
| COL1A1-E50SR |  | GAGAGGGCACTATGGCCTGGC |
