# Supplementary material for: Expanding the clinical spectrum of COL1A1 mutations in different forms of glaucoma
Source: Orphanet J Rare Dis. 2016 Aug 2;11:108. doi: 10.1186/s13023-016-0495-y (PMC4970237; doi:10.1186/s13023-016-0495-y)

**Additional file 2**

**Sequence chromatograms of the variants identified in *COL1A1* gene in three different patients**


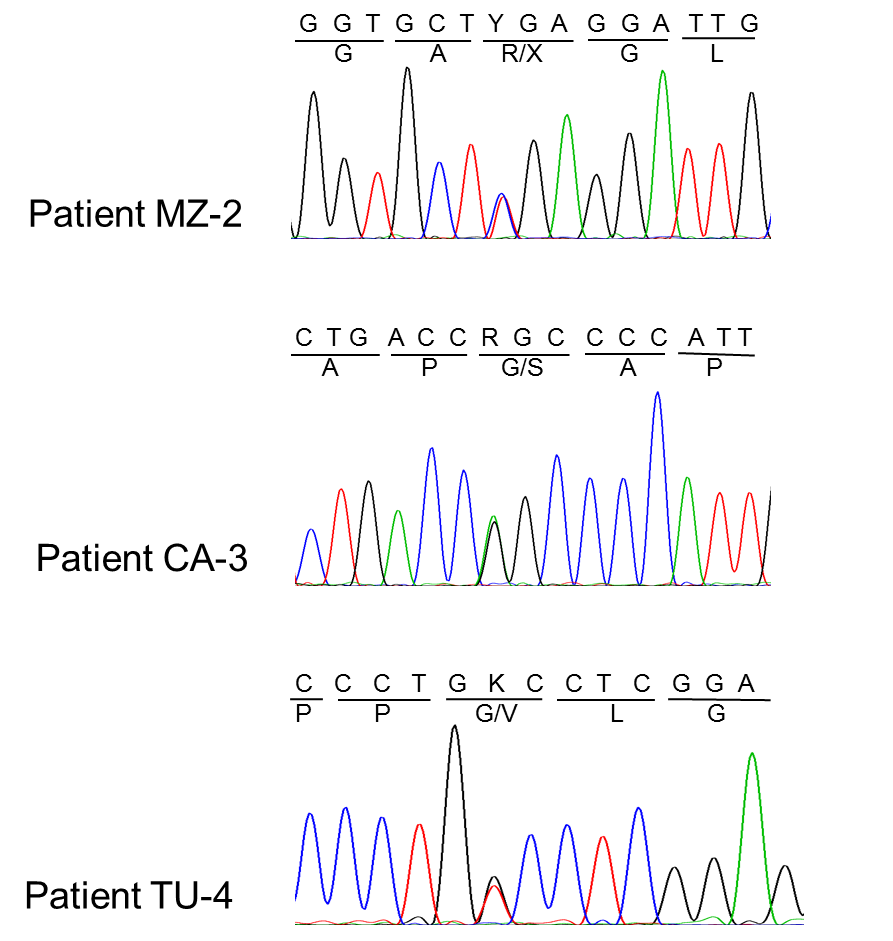

Supplement: Additional file 2: — Sequence chromatograms of the variants identified in COL1A1 gene in three different patients: MZ-2, CA-3 and TU-4. (DOCX 129 kb) [file 13023_2016_495_MOESM2_ESM.docx]
